# Mitochondrial ROS Production at Complexes I and III in Human Myocardium and Skeletal Muscle: A Distinct Pattern Compared with Rat Tissue

**DOI:** 10.3390/cells15090830

**Published:** 2026-05-01

**Authors:** Ivan Mihanovic, Jasna Marinovic, Cristijan Bulat, Bruno Luksic, Zlatko Marovic, Marko Ljubkovic

**Affiliations:** 1 Department of Physiology, University of Split School of Medicine, Soltanska 2A, 21 000 Split, Croatia; ivan.mihanovic@kbsplit.hr (I.M.); jasna.marinovic@mefst.hr (J.M.); 2 Department of Infectious Diseases, University Hospital of Split, Spinciceva 1, 21 000 Split, Croatia; 3 Division of Cardiosurgery, University Hospital of Split, Spinciceva 1, 21 000 Split, Croatia; cristijan.bulat.st@gmail.com (C.B.); zlatko09@gmail.com (Z.M.); 4 Department of Surgery, Division of Orthopaedics and Traumatology, University Hospital of Split, Spinciceva 1, 21 000 Split, Croatia; bruno.luksic@kbsplit.hr

**Keywords:** human myocardium, skeletal muscle, mitochondrial reactive oxygen species, complex I, complex III, mitochondrial ROS hierarchy

## Abstract

**Highlights:**

**What are the main findings?**

- Direct comparison of mitochondrial ROS production in human and rat cardiac and skeletal muscle revealed species-specific differences in dominant ROS sources: complex I (RET) predominated in humans, whereas complex III (IIIQo) was the major source in rats.
- When normalized to respiratory activity, human mitochondria exhibited a greater relative capacity for ROS production at complex I, while ROS generation at complex III remained comparatively lower than in rat tissues.

**What are the implications of the main findings?**

- These results challenge the assumption that ROS production patterns observed in rodent models directly translate to human physiology.
- Species-specific differences in mitochondrial ROS generation may influence the effectiveness of cardioprotective strategies targeting oxidative stress, highlighting the need for human-focused approaches.

**Abstract:**

Mitochondrial reactive oxygen species (ROS) play a central role in cardiac ischemia/reperfusion injury, heart failure, and arrhythmogenesis, while also serving essential signaling functions under physiological conditions. Among the eleven identified mitochondrial ROS-producing sites, complexes I and III are considered the major contributors, particularly under conditions of impaired electron flow. However, much of the existing knowledge comes from rodent models or cultured cells and is often assumed to apply to humans. Here, ROS production from complexes I and III was measured directly in human myocardial and skeletal muscle biopsies and compared with corresponding rat tissues under identical experimental conditions. Hydrogen peroxide generation was quantified using Amplex UltraRed, with simultaneous monitoring of mitochondrial respiration using a Clark-type oxygen electrode. Across all examined mechanisms—reverse and forward electron transport at complex I and the ubiquinol oxidation site of complex III, rat tissues produced more ROS than human tissues, consistent with their higher respiratory rates. However, the dominant ROS-producing sites differed: in rats, complex III was the primary source, whereas in human tissues the highest ROS production occurred during reverse electron transport at complex I. When normalized to respiration, human tissues showed relatively greater ROS generation at complex I but markedly lower production at complex III. These direct measurements of mitochondrial ROS production in human myocardium provide new insight into cardiac redox physiology and may explain the limited clinical translation of cardioprotective strategies targeting mitochondrial ROS production, such as interventions aimed at modulating reperfusion injury or preconditioning.

## 1. Introduction

Cardiovascular diseases remain the leading cause of mortality worldwide, with ischemia/reperfusion (IR) injury, heart failure, and vascular dysfunction representing major clinical challenges. Reactive oxygen species (ROS), of which superoxide (O_2_•^−^) and hydrogen peroxide (H_2_O_2_) are the most frequently produced, are important mediators in these conditions [1]. At excessive levels, they contribute to cardiomyocyte death, contractile dysfunction, and arrhythmias [2,3], while at moderate levels, they play essential roles in normal physiology, acting as signaling molecules in processes such as adaptation to stress and regulation of cellular function [4,5,6]. Beyond the cardiovascular system, ROS are implicated in cancer, diabetes, neurodegeneration, aging, host defense, and even sleep regulation [7,8,9,10,11], underscoring their wide-ranging physiological relevance.

Mitochondria are the dominant source of ROS in cardiomyocytes [12]. Among the eleven known mitochondrial sites capable of producing ROS, complexes I and III of the electron transport chain (ETC) are considered the principal contributors [13,14]. Complex I (NADH-ubiquinone oxidoreductase) produces high amounts of ROS during reverse electron transport (RET), when electrons are driven from ubiquinol back into complex I by a high proton motive force [15,16]—a condition that arises during early reperfusion after ischemia [17]. Substantial amount of ROS can also be generated during forward electron transport (FET), especially when NADH levels are high and the downstream electron flow is blocked [18,19]. Complex III (ubiquinol-cytochrome c oxidoreductase) generates ROS mainly at the ubiquinol oxidation site (III_Qo_), particularly when the Qi-site is inhibited [20,21], as might occur during prolonged ischemia [22]. In general, mitochondrial ROS generation increases sharply when electron carriers become excessively reduced and the respiratory chain is congested.

Despite the recognized importance of these mechanisms, most current knowledge is derived from rodent models and cell systems. Direct evidence from human cardiac tissue remains scarce, and mitochondrial ROS production has not previously been systematically characterized in human left ventricular myocardium.

In this study, we examined mitochondrial ROS generation at complexes I and III in human heart and skeletal muscle, alongside measurements of mitochondrial respiration. Using the same experimental conditions, we also analyzed the corresponding rat tissues to enable direct comparison. This approach identified species-specific differences in ROS production between complexes I and III that are relevant when interpreting data from animal models, both in studies of human cardiovascular disease and in investigations of physiological redox regulation.

## 2. Materials and Methods

### 2.1. Ethical Considerations

All procedures were reviewed and approved by the Ethics Committees of the University Hospital Split (2181-147/01/06/M.S.-21-02) and the University of Split School of Medicine (2181-198-03-04-20-0080), in accordance with the Declaration of Helsinki. Written informed consent was obtained from all participants, prior to their enrollment. Patients were recruited as part of the observational study registered at http://www.clinicaltrials.gov (accessed on 28 February 2026) under the identification number NCT03179137.

### 2.2. Human Cardiac Left Ventricular Biopsies

Twelve hemodynamically stable patients (9 males) undergoing coronary artery bypass grafting (CABG) surgery at the University Hospital Split were included in the study. Their mean age was 64 ± 6 years, and the main clinical characteristics are shown in Table 1. Excluded were emergency cases, patients with left ventricular ejection fraction (LVEF) < 40%, type 1 diabetes, concomitant valve replacement, or severe renal, hepatic, or pulmonary disease. Myocardial biopsies were obtained from the anteroseptal region of the left ventricle (LV), before graft placement, using 14-gauge disposable needles (Magnum Reusable Core Biopsy Instrument; Bard Biopsy Systems, Tempe, AZ, USA) [23]. All surgeries were performed “off-pump”, without the use of cardiopulmonary bypass or cardioplegia, and no complications evidently related to the LV biopsy procedure were observed. The tissue was immediately placed in ice-cold biopsy preservation solution (BIOPS: 7.23 mM K_2_EGTA, 2.77 mM CaK_2_EGTA, 50 mM K-methane sulfonate, 6.56 mM MgCl_2_, 5.77 mM Na_2_ATP, 15 mM Na_2_Phoshocreatine, 20 mM imidazole, 20 mM taurine, 0.5 mM dithiothreitol, pH adjusted to 7.1 at 0 °C with KOH). Samples were kept on ice and transferred to the laboratory within 15 min for preparation of tissue homogenates.

### 2.3. Human Skeletal Muscle Biopsies

Skeletal muscle tissue was obtained from the vastus lateralis muscle in six healthy male volunteers [24]. They reported recreational sports activity once or twice a week and abstained from intense exercise at least two days before the biopsy procedure. Following skin sterilization and subcutaneous local anesthesia with 2% lidocaine, a 5 mm-long incision was made using the tip of a scalpel, cutting through the skin and muscle fascia. The muscle samples were collected using a 14-gauge disposable needle (Bard Mission Disposable Core Biopsy Instrument), inserted through the incision perpendicular to the muscle fibers. Samples weighing 8–10 mg were immediately processed for homogenization.

### 2.4. Experimental Animals and Tissues (Heart and Skeletal Muscle)

Adult male Sprague-Dawley rats, weighing 250–280 g, with free access to water and standard laboratory diet, were anesthetized with 4% isoflurane, followed by an intraperitoneal injection of ketamine (Ketaminol, 90 mg/kg, Intervet International B.V., Boxmeer, The Netherlands) and xylazine (Xylapan, 8 mg/kg, Vetoquinol, Lure, France). Full anesthesia was confirmed by the absence of the corneal reflex. Once anesthetized, tissue samples were excised from either the left cardiac ventricle (n = 15) [25] or the right soleus muscle (n = 9) [26], and homogenates prepared.

### 2.5. Tissue Homogenates

Immediately after harvesting, all collected tissue samples (from both humans and rats) were placed in ice-cold BIOPS solution and carefully cleaned of any connective and fat tissue under a magnifier. The wet weight of each sample was precisely measured, after which the BIOPS solution was washed off using mitochondrial respiration buffer (MiR05: 1 g/L bovine serum albumin, 110 mM sucrose, 20 mM HEPES, 20 mM taurine, 10 mM KH_2_PO_4_, 0.5 mM EGTA, 60 mM lactobionic acid, 3 mM MgCl_2_, pH adjusted to 7.1 at 30 °C with KOH), supplemented with NADH-producing substrates (5 mM pyruvate and 0.5 mM malate). Each sample was homogenized using a semi-automated mechanical grinder (PBI Shredder SG3 system, Pressure Biosciences, Canton, MA, USA) [27], and every resulting homogenate was diluted with MiR05 and divided into aliquots which were analyzed in parallel chambers using different substrate protocols.

### 2.6. Assessment of Respiration and ROS Production

The rates of tissue oxygen consumption and H_2_O_2_ production were simultaneously measured using a Clark-type electrode with an attached fluorescence module (O2k-FluoRespirometry system, Oroboros Instruments, Innsbruck, Austria) [28]. All experiments were conducted at 30 °C, and oxygen concentration in the chambers was maintained between 200–220 µM to minimize the effect of oxygen levels on measured parameters. Oxygen concentration was determined by calibration of the system at air saturation at 30 °C, based on atmospheric pressure and the solubility factor of the MiR05 respiration buffer. Substrates and inhibitors were added in small volumes relative to the chamber volume, minimizing potential effects on oxygen solubility, and preliminary experiments confirmed that ROS production rates remained stable at measured oxygen concentrations above ~200 µM. The concentrations of chemicals used in the experiments are listed in Table 2.

#### 2.6.1. Respiration

Respiration rates were measured using complementary protocols in separate experimental chambers, with homogenates from each sample aliquoted accordingly. In the first protocol, LEAK respiration supported by complex I (LEAK CI) was measured in the presence of pyruvate, malate, and glutamate in the absence of ADP. LEAK respiration represents the oxygen consumption required to compensate for proton leak across the inner mitochondrial membrane in the absence of ATP synthase activity. Subsequently, a saturating concentration of ADP was added to assess oxidative phosphorylation (OxPhos CI), followed by addition of succinate to determine maximal oxidative phosphorylation capacity supported by both complexes I and II (OxPhos CI + CII).

In the second protocol, pyruvate, malate, glutamate, and succinate were present from the beginning to assess LEAK respiration supported by both complexes I and II (LEAK CI + CII). Here, ADP was omitted and oligomycin was added to inhibit ATP synthase. These conditions were used for simultaneous measurements of respiration and ROS production via RET.

#### 2.6.2. ROS Production

Amplex UltraRed assay (Thermo Fisher Scientific, Waltham, MA, USA), which detects H_2_O_2_, was used to assess ROS production rates. The measured H_2_O_2_ signal reflects the combined production of superoxide and hydrogen peroxide, since superoxide is rapidly converted to hydrogen peroxide by both endogenous and added SOD [9,29,30].

Tissue homogenates were incubated with 2.5 μM Amplex UltraRed, 5 U/mL SOD, and 1 U/mL horseradish peroxidase (HRP). The formation of a resorufin-like fluorescent product, which occurs in a 1:1 ratio with H_2_O_2_ production, was detected at 587 nm following excitation at 525 nm [30,31]. In each experiment, background fluorescence (measured in the absence of tissue) was subtracted, and the fluorescent signal was calibrated using freshly prepared H_2_O_2_ standard (0.1 μM). Normalization of ROS production to oxygen consumption was used to account for differences in mitochondrial content between tissues and species.

Three canonical modes of mitochondrial ROS production were evaluated; (1) Production at complex I induced by reverse electron transport (RET CI); (2) Production at complex I induced by inhibition of ubiquinone-binding site (I_Q_) during forward electron transport (FET CI); and (3) Production at the ubiquinol-oxidation site at complex III (III_Qo_ site) induced by inhibition of III_Qi_ site (inner or negative side site) [14].

##### RET CI

**Figure 1:**
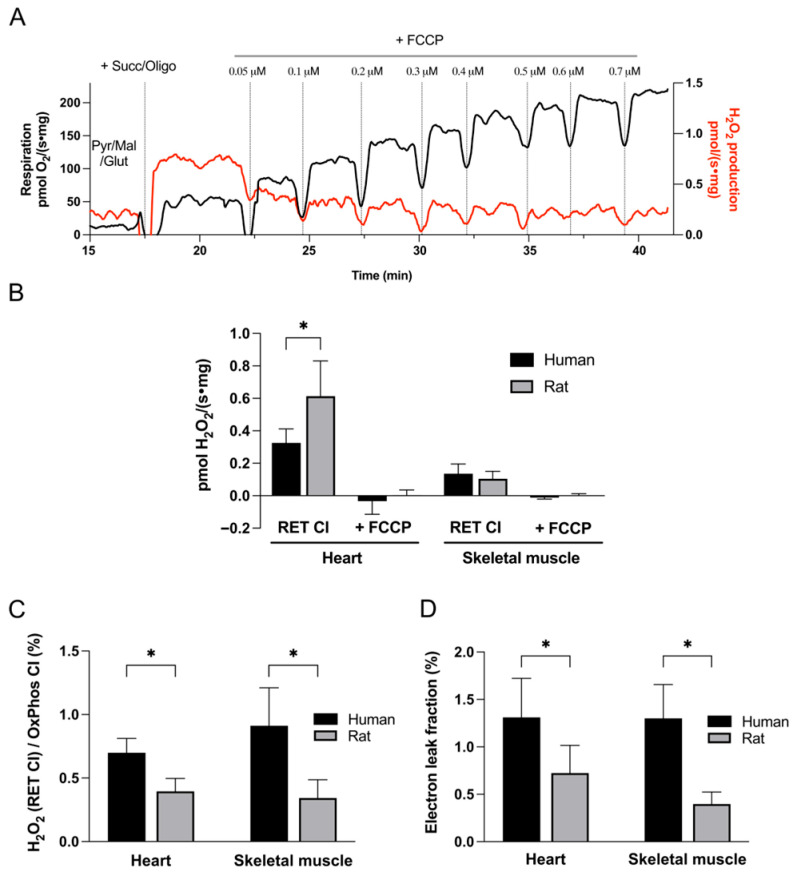
**Mitochondrial ROS production via reverse electron transport at complex I (RET CI).** (**A**) Representative recording of simultaneous measurements of mitochondrial respiration (black) and ROS production (red) in rat myocardium. Tissue homogenate was initially incubated with pyruvate, malate, and glutamate (Pyr/Mal/Glut) under non-phosphorylating conditions (no ADP). Subsequent addition of succinate and oligomycin (Succ/Oligo) established conditions favoring reverse electron transport, characterized by a high proton motive force and a reduced ubiquinone pool, resulting in an increase in ROS production. This signal was abolished by FCCP. Additions cause brief transient deflections in the signal due to injection, after which stable steady-state rates were used for analysis. (**B**) Summary data showing absolute rates of ROS production under RET CI conditions. (**C**) ROS production normalized to complex I-supported oxidative phosphorylation (OxPhos CI). (**D**) Electron leak fraction, expressed as the proportion of total respiratory electron flow diverted to ROS formation. Data are presented as mean ± SD for human heart (n = 12), rat heart (n = 15), human skeletal muscle (n = 6), and rat skeletal muscle (n = 9). * *p* < 0.05.

**Figure 2:**
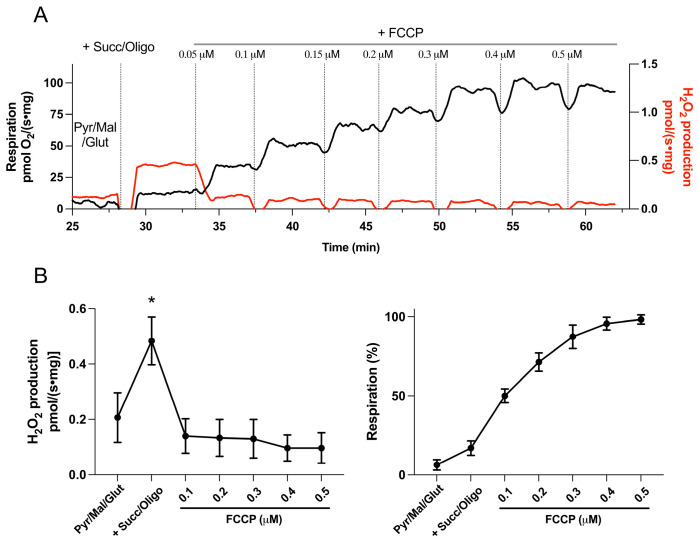
**Dependence of RET CI-induced ROS production on proton motive force (Δp) in human heart mitochondria.** (**A**) Representative recording of mitochondrial respiration (black) and H_2_O_2_ production (red) in human myocardium. Tissue homogenate was incubated with pyruvate, malate, and glutamate (Pyr/Mal/Glut) under non-phosphorylating conditions. Addition of succinate and oligomycin (Succ/Oligo) established conditions for reverse electron transport and stimulated ROS production. Subsequent addition of the protonophore FCCP abolished ROS production and increased oxygen consumption. (**B**) Summary data showing ROS production (left) and respiration (right; n = 5). Addition of 0.1 µM FCCP abolished RET CI-driven ROS production, despite only partial dissipation of Δp, as indicated by an incomplete increase in respiration. Higher FCCP concentrations further increased respiration without additional effects on ROS production. * *p* < 0.05 vs. LEAK CI and FCCP.

Tissue homogenates were incubated with NADH-generating substrates (pyruvate, malate, and glutamate) until a stable baseline fluorescence signal was achieved. RET conditions were then established by addition of succinate at a saturating concentration, generating a reduced ubiquinone pool and high proton motive force (Δp). Oligomycin was present to inhibit ATP synthase, and no ADP was added. The resulting increase in fluorescence signal was taken as a measure of ROS production via RET at complex I (Figure 1A and Figure 2A). This effect was subsequently abolished by a protonophore FCCP, confirming its dependence on Δp [15].

The so-called electron leak refers to the proportion of electrons that, instead of progressing through the ETC to fully reduce oxygen to water, prematurely reduce O_2_ to form reactive species such as superoxide. This term should not be confused with LEAK respiration, which denotes non-phosphorylating oxygen consumption measured in the absence of added ADP. We calculated the electron leak fraction as follows: the rate of H_2_O_2_ production was multiplied by 2 (since 2 electrons are required for partial reduction of oxygen to H_2_O_2_), and this value was divided by total concomitant oxygen consumption (LEAK CI + CII) multiplied by 4 (reflecting the 4 electrons needed for complete O_2_ reduction to water) [32].

##### FET CI

**Figure 3:**
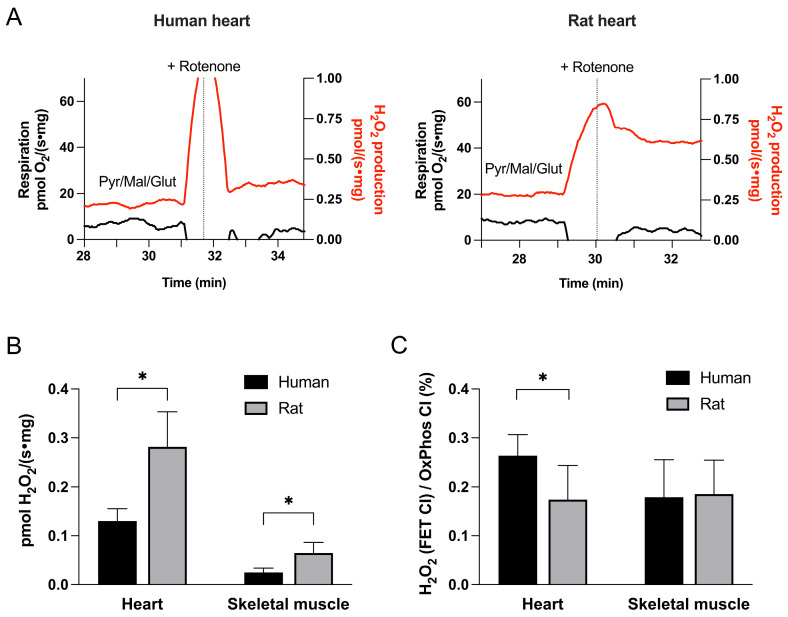
**Mitochondrial ROS production via forward electron transport at complex I (FET CI).** (**A**) Representative recordings from human (left) and rat (right) myocardium. ROS production (red traces) was initially recorded in the presence of NADH-generating substrates pyruvate, malate, and glutamate (Pyr/Mal/Glut) under non-phosphorylating conditions (no ADP). Subsequent addition of rotenone, an inhibitor of the ubiquinone-binding site of complex I (I_Q_), resulted in an increase in ROS signal. Additions cause brief transient deflections in the signal due to injection, after which steady-state rates were used for analysis. (**B**) Summary data showing absolute rates of ROS production across human and rat heart and skeletal muscle tissues. (**C**) ROS production normalized to complex I-supported oxidative phosphorylation (OxPhos CI). Data are presented as mean ± SD for human heart (n = 12), rat heart (n = 15), human skeletal muscle (n = 6), and rat skeletal muscle (n = 9). * *p* < 0.05.

Tissue homogenates were incubated with NADH-generating substrates (pyruvate, malate, and glutamate) in the absence of ADP until a stable fluorescence signal was obtained. ROS production via FET at complex I was then induced by addition of rotenone, an inhibitor of the I_Q_ site (Figure 3A). The increase in fluorescence signal following rotenone addition was taken as a measure of FET CI-derived ROS production [18].

##### IIIQo Site

**Figure 4:**
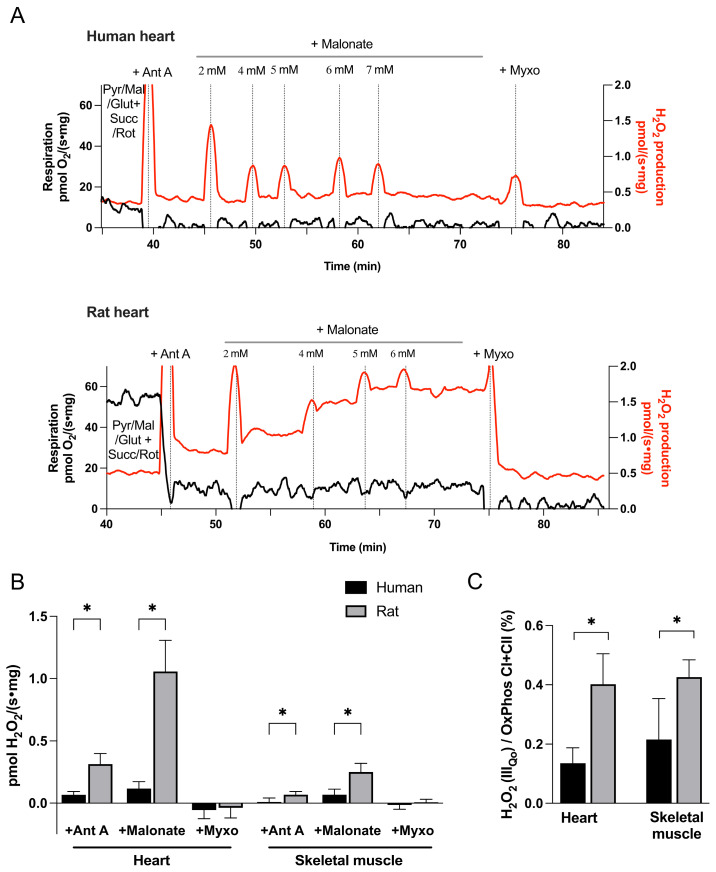
**ROS production at the ubiquinol oxidation site of complex III (III_Qo_).** (**A**) Representative recordings from human (**top**) and rat (**bottom**) heart homogenates. ROS production (red traces) was initially measured under non-phosphorylating conditions in the presence of pyruvate, malate, and glutamate (Pyr/Mal/Glut). Complex I was inhibited by rotenone, and succinate was added to supply electrons via complex II (Succ/Rot). Addition of the III_Qi_ site inhibitor antimycin A (Ant A) stimulated ROS production at the III_Qo_ site, with a markedly greater response in rat compared to human tissue. Stepwise addition of malonate further increased ROS production, particularly in rat, whereas myxothiazol (Myxo), an inhibitor of the III_Qo_ site, abolished the antimycin A- and malonate-induced increase in ROS production. (**B**) Summary data showing absolute rates of ROS production at the III_Qo_ site in human and rat heart and skeletal muscle. (**C**) ROS production at the III_Qo_ site normalized to oxidative phosphorylation supported by complexes I and II (OxPhos CI + CII). Data are presented as mean ± SD for human heart (n = 12), rat heart (n = 15), human skeletal muscle (n = 6), and rat skeletal muscle (n = 9). * *p* < 0.05.

Tissue homogenates were incubated with NADH-generating substrates (pyruvate, malate, and glutamate) under non-phosphorylating conditions (no ADP). Complex I was inhibited by rotenone and succinate was added to provide electrons to the ubiquinone pool via complex II. ROS production at the complex III_Qo_ site was then stimulated by addition of antimycin A, an inhibitor of the III_Qi_ site (Figure 4A). To further optimize ROS production at the III_Qo_ site, malonate was added stepwise to partially inhibit complex II and achieve an optimal ubiquinol/ubiquinone ratio [20,21]. The resulting increase in fluorescence signal was attributed to ROS production at the III_Qo_ site and was abolished by addition of myxothiazol.

### 2.7. Chemicals

All chemicals used were purchased from Sigma-Aldrich (St. Louis, MO, USA), unless stated otherwise.

### 2.8. Statistical Analysis

Statistical analysis was performed using GraphPad Prism 10. Normality of data distribution for all variables was assessed using the Shapiro–Wilk test. Comparisons of mitochondrial respiration and H_2_O_2_ production between tissues or species were tested using either a one-way ANOVA for independent samples with Šidák’s post hoc correction for multiple comparisons, or an unpaired Student’s *t*-test. ROS production measured at different mitochondrial sites within the same tissue was compared using a one-way repeated-measures ANOVA, followed by Tukey’s post hoc test to adjust for multiple comparisons. Data are presented as mean ± standard deviation (SD). A *p*-value < 0.05 was considered statistically significant.

## 3. Results

### 3.1. Mitochondrial Respiration

Mitochondrial respiratory rates, expressed as oxygen consumption per milligram of tissue, under different substrate conditions are summarized in Table 3. Overall, respiration was substantially higher in rat compared with human tissues. In the heart, oxygen consumption was approximately threefold greater in rats than in humans, and in skeletal muscle it was about 2.5-fold higher in rats. Within each species, cardiac tissue exhibited higher respiration than skeletal muscle, showing roughly a 2.5-fold difference in humans and nearly a fourfold difference in rats.

### 3.2. ROS Production

#### 3.2.1. RET CI-Induced ROS Production

The highest rate of RET CI-driven ROS production was observed in rat heart, approximately twice as high as that detected in human heart tissue (Figure 1B). This result aligns with the higher respiration rates recorded in rat myocardium. Despite significantly greater respiratory rates in rat skeletal muscle relative to human, there were no significant species differences in skeletal muscle RET CI-derived ROS.

To account for differences in mitochondrial content between tissues and species, ROS production rates were also expressed relative to oxygen consumption, providing an index of ROS generation per unit of electron flux. When ROS production was normalized to mitochondrial respiration, a different pattern emerged: human mitochondria exhibited a higher propensity for ROS generation via this mechanism compared to rat mitochondria. As shown in Figure 1C, ROS production via RET CI, expressed relative to complex I-mediated respiration (OxPhos CI), was nearly twofold higher in human than in rat heart, and approximately threefold higher in human than in rat skeletal muscle. Consistent with this, the calculated electron leak fraction (Figure 1D)—representing the percentage of electrons that are diverted to ROS formation—was almost doubled in human versus rat heart (1.31 ± 0.41% vs. 0.72 ± 0.29%), and nearly threefold higher in human compared with rat skeletal muscle (1.30 ± 0.36% vs. 0.39 ± 0.13%).

It is well established that a high Δp is required for RET CI to occur [15], and addition of the protonophore FCCP fully abolished ROS production via this mechanism (Figure 1B). The sensitivity of RET CI to changes in Δp in the human heart is further demonstrated in Figure 2. The application of a low dose of FCCP (0.1 μM) caused only partial mitochondrial depolarization, as reflected by a moderate increase in respiration (approximately 50% of the maximal oxygen consumption rate), yet it completely abolished RET CI-derived H_2_O_2_ generation (Figure 2B).

#### 3.2.2. FET CI-Induced ROS Production

The rate of ROS production via forward electron transport at complex I, measured in the presence of rotenone to inhibit electron flow beyond complex I, was more than twice as high in rat mitochondria compared to human, in both heart and skeletal muscle tissues (Figure 3B). However, when normalized to complex I-mediated respiration (Figure 3C), human heart mitochondria showed higher relative ROS generation—similar to the pattern observed for RET CI-induced ROS. This species-specific difference was not observed in skeletal muscle.

#### 3.2.3. III_Qo_ Site ROS Production

The rate of ROS production at the III_Qo_ site, with the III_Qi_ site inhibited by antimycin A, was significantly higher in rat heart and skeletal muscle compared to the corresponding human tissues (Figure 4B). This difference became even more pronounced after addition of malonate: in rat tissues, H_2_O_2_ production increased three- to fourfold, consistent with previous reports [20]. In contrast, human tissues had a very modest response to malonate. At the peak of malonate-stimulated ROS generation, the H_2_O_2_ production rate in rat heart was nearly nine-fold higher than in human heart (1.05 ± 0.25 vs. 0.12 ± 0.05 pmol/(mg·s)), and approximately 3.5-fold higher in rat compared to human skeletal muscle (0.25 ± 0.07 vs. 0.07 ± 0.04 pmol/(mg·s)). In all examined tissues, ROS generation at the III_Qo_ site was completely abolished by myxothiazol (the site inhibitor).

When normalized to oxidative phosphorylation capacity, ROS production from the III_Qo_ site remained markedly higher in rat tissues (Figure 4C). This is in contrast with our findings for RET CI and FET CI, where relative ROS production was greater in human tissues.

#### 3.2.4. Hierarchy of ROS-Producing Mechanisms

**Figure 5:**
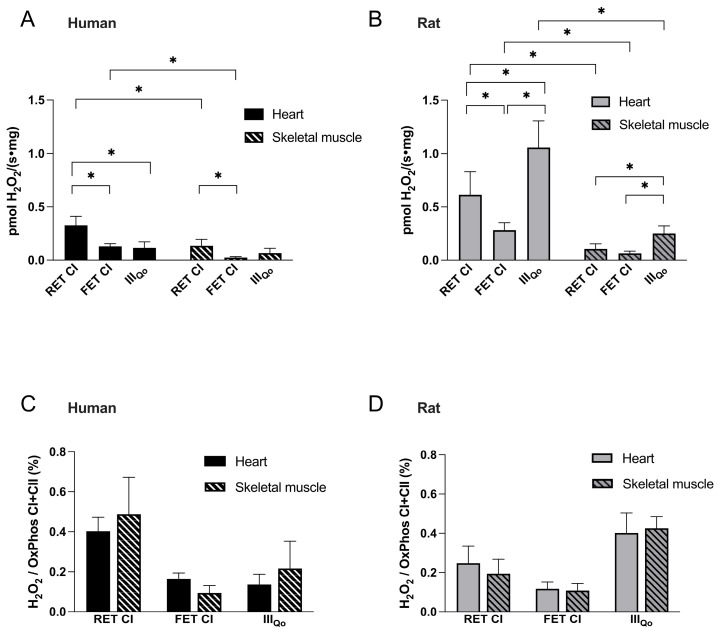
**Hierarchies of mitochondrial ROS production in human and rat tissues.** (**A**,**B**) Comparison of ROS production through the three major mitochondrial mechanisms (RET CI, FET CI, and III_Qo_) in human and rat heart and skeletal muscle, respectively. (**C**,**D**) Relative ROS production (normalized to respiration) in heart versus skeletal muscle of human and rat, respectively. Data are mean ± SD from human heart (n = 12), rat heart (n = 15), human skeletal muscle (n = 6) and rat skeletal muscle (n = 9). * *p* < 0.05.

When the three ROS-generating mechanisms were compared within each tissue, distinct hierarchies emerged. In human heart and skeletal muscle, the highest ROS production was observed during RET at complex I, whereas ROS generation at the III_Qo_ site was markedly lower and comparable to that observed during FET CI (Figure 5A). In contrast, rat tissues displayed a different pattern, with complex III (III_Qo_ site) representing the predominant source of ROS, followed by RET CI and, to a lesser extent, FET CI (Figure 5B).

In both human and rat tissues, absolute ROS production via all three mechanisms was greater in the heart than in skeletal muscle, consistent with the higher respiratory activity in cardiac tissue. However, this inter-organ difference disappeared when ROS production was normalized to respiration (Figure 5C,D).

## 4. Discussion

In this study, we directly quantified mitochondrial superoxide and H_2_O_2_ production in human and rat cardiac and skeletal muscle, focusing on three canonical mechanisms of mitochondrial ROS generation: reverse electron transport at complex I (RET CI), forward electron transport at complex I (FET CI), and production at the outer quinol-binding site of complex III (III_Qo_ site). By employing a uniform methodological approach across tissues, we identified yet unrecognized and potentially relevant interspecies differences in mitochondrial ROS profiles.

Our results demonstrate that, in both heart and skeletal muscle, rats exhibit greater absolute ROS production via all three tested mechanisms compared to humans (the only exception being RET CI in skeletal muscle). This likely stems from markedly higher mitochondrial respiratory activity observed in rats, consistent with their greater mitochondrial density and metabolic rate [33,34,35]. Higher respiration rates have been widely associated with greater ROS generation, as observed in various physiological and pathological contexts. This concept is central to “the rate of living” theory, which postulates that smaller species with higher metabolic rates experience greater oxidative stress, contributing to shorter lifespans [36,37]. Moreover, within a species, physiological adaptations that increase maximal oxygen consumption also result in greater capacity for ROS production. An example is skeletal muscle during adaptation to endurance training [38,39]. Finally, at the organ level, tissues with higher energetic demands tend to exhibit greater oxygen consumption and ROS production. This has been demonstrated by comparing mitochondria from different organs, such as the brain versus liver [40] or cardiac versus skeletal muscle [30,41].

In rats, the hierarchy of mitochondrial ROS production mechanisms studied here followed the expected pattern: the highest output originated from complex III (III_Qo_), followed by RET CI, and then FET CI. These observations are consistent with prior studies in rodents demonstrating a prominent role of complex III in mitochondrial ROS production [14,42]. In contrast, human cardiac and skeletal muscle tissues displayed a different profile: RET CI was the dominant source of ROS, while production at the III_Qo_ site was considerably lower and comparable to that seen in FET CI. This striking difference between species is a novel finding of this study.

Several factors may underlie the observed interspecies differences. One possibility is that humans inherently produce less ROS at the III_Qo_ site under antimycin-inhibited conditions than rats. Alternatively, the topology of ROS release may differ. Complex III is unique in that it can release superoxide and H_2_O_2_ both into the mitochondrial matrix and into the intermembrane space, depending on the direction and redox state of the electron carriers. This bidirectional release has been demonstrated in studies specifically addressing ROS directionality at the III_Qo_ site [43,44]. In humans, a greater proportion of ROS generated at III_Qo_ could be directed into the mitochondrial matrix, where superoxide is rapidly converted to H_2_O_2_ by matrix SOD. This, together with directly produced H_2_O_2_, may then be neutralized by matrix antioxidant systems before diffusing out of the mitochondria and being detected by the Amplex UltraRed assay, which reports only extramitochondrial H_2_O_2_ [44,45]. Consequently, this could lead to an apparent reduction in measurable ROS and underestimation of total III_Qo_ ROS production in human tissues.

Although absolute ROS production rates were consistently higher in rat tissues, our data reveal that human mitochondria generate more ROS at complex I relative to their respiratory activity, both via RET and FET. This suggests a greater propensity for electron leak at complex I in human mitochondria per unit of respiratory flux. One potential explanation is variation in the structural and supramolecular organization of complex I. Subtle alterations in its amino acid composition have been shown to significantly affect function; for example, a single-point mutation in complex I in ND6-P25L mice abolishes RET and confers protection against IR injury [46]. In addition to primary structure, the organizational state of complex I—specifically the balance between free enzyme and its incorporation into supercomplexes with complexes III and IV—can influence ROS production. This has been demonstrated in neurons and astrocytes, where a higher proportion of free complex I is associated with lower respiration and increased ROS generation [47]. Although direct comparisons between human and rat mitochondria are limited, differences in the degree of supercomplex assembly or stability could plausibly contribute to the higher relative complex I-derived ROS production observed in human tissues in the present study. These considerations provide a mechanistic framework linking our findings to known determinants of mitochondrial ROS generation.

Another possible explanation for the higher complex I-derived ROS production per unit of respiration observed in human tissues is that rat mitochondria may possess more effective antioxidant defenses, such as SODs, catalase and the thioredoxin and glutathione antioxidant systems [48,49,50], compared to those of humans. Because the Amplex UltraRed assay detects H_2_O_2_ that escapes mitochondrial scavenging systems, the measured signal reflects the balance between ROS production and detoxification. Differences in antioxidant capacity could therefore influence the apparent rates of ROS release detected in our experiments. However, direct comparative studies between rats and humans are lacking. Insights can be drawn from studies in other species: for example, naked mole-rats (NMRs) exhibit two- to fivefold greater mitochondrial matrix antioxidant capacity in skeletal muscle and heart compared to mice [51]. These findings support the hypothesis that enhanced antioxidant capacity, rather than reduced ROS production per se, may characterize long-lived species. Consistent with this idea, it could be expected that human mitochondria, relative to their respiratory activity, are equipped with a more robust ROS detoxification system than those of rats. Nonetheless, considerable species- and tissue-specific variability in antioxidant capacity has been reported [45], and in the absence of direct comparisons, such conclusions remain speculative.

The relatively high rate of complex I-derived ROS generation in humans becomes especially relevant in IR injury, where RET-driven ROS play a central role [17]. During ischemia, succinate accumulates in tissues and, upon reperfusion, it is rapidly oxidized, driving a burst of ROS production via RET at complex I [17,52]. This mechanism has been extensively validated in animal studies. Moreover, interventions that limit succinate oxidation, such as succinate dehydrogenase inhibitors like malonate, have successfully reduced IR injury in preclinical models [53]. In humans, a burst of ROS has been demonstrated following coronary artery revascularization during CABG surgery [54]. In addition, metabolomic profiling of STEMI patients revealed elevated succinate levels in coronary-sinus and arterial blood during reperfusion, which correlated with the extent of myocardial injury assessed by imaging [55]. These findings support the relevance of RET mechanism in humans (substrate that drives RET is present).

Nevertheless, translation of cardioprotective strategies targeting mitochondrial ROS—such as ischemic preconditioning or pharmacological modulation of succinate oxidation—has been inconsistent in clinical settings [56,57]. One possible explanation is that these approaches were largely developed and validated in animal models, where the dominant sources of ROS differ from those observed in human tissues. In particular, the prominence of complex III-derived ROS in rodents versus the predominance of RET-driven complex I ROS in humans may lead to different responses to interventions targeting specific sites or mechanisms of ROS production. Recognizing this divergence highlights the importance of our finding that RET at complex I represents the highest-capacity ROS-producing mode in human tissues and underscores the need for human-focused research into IR pathophysiology.

Interestingly, despite humans exhibiting greater relative ROS production at complex I, under RET and FET conditions, our data reveal that ROS generation from the III_Qo_ site remains consistently lower in humans in both heart and skeletal muscle—even after normalization to oxidative phosphorylation capacity. While complex I-derived, RET-induced ROS is considered the primary driver of IR injury, ROS originating from complex III—particularly at the III_Qo_ site—has also been implicated in the pathogenesis of several chronic conditions, including heart failure, neurodegeneration, and aging. For example, ischemia-damaged complex III was highlighted as a key site of ROS generation after permeability transition pore (PTP) opening in rabbit cardiac mitochondria [22]. In a cellular model of Parkinson’s disease, complex III-generated ROS was found to elicit oxidative damage, exacerbating mitochondrial dysfunction and potentially contributing to the disease’s pathophysiology [58]. Further, complex III-derived ROS in macrophages was found to promote interleukin-10 production via AP-1/c-Fos, thereby suppressing antitumor immunity; inhibition of this pathway improved survival in mouse melanoma models [59].

Although ROS production at III_Qo_ site is typically observed in the presence of III_Qi_ site inhibitors, such as antimycin A, it has been demonstrated that under conditions of elevated mitochondrial membrane potential, the III_Qo_ site alone can generate ROS at rates comparable to those observed during enzyme inhibition [60]. Given the high ROS-generating capacity of complex III in rodent models, this finding implicates complex III as a potentially significant source of ROS in vivo, contributing to both physiological signaling and pathophysiological oxidative stress. The lower ROS production at the III_Qo_ site in humans could therefore represent a protective adaptation, possibly contributing to greater resistance to chronic ROS-mediated damage in tissues with high oxidative metabolism.

Our findings that rats and humans differ in various aspects of mitochondrial ROS production, including the hierarchy of production sites and ROS generation relative to mitochondrial respiration, might have implications for the use of rat models in human research. Given that the pattern and relative contribution of individual ROS-generating mechanisms differ between rat and human tissues, extrapolation from rodents to humans should be approached with caution, particularly when investigating oxidative stress and redox-sensitive signaling pathways. Since rat models are extensively used to study mitochondrial oxidative stress and its role in cardiac pathology—including IR injury and heart failure—these differences raise concerns regarding their translational relevance. This may be particularly important for therapeutic strategies that target specific mitochondrial ROS sources, as their efficacy could depend on species-specific differences in the dominant sites and mechanisms of ROS production.

A key limitation of our study is the reliance on H_2_O_2_ efflux as a proxy for superoxide production, which is affected by both production and scavenging. In addition, the use of tissue homogenates does not preserve mitochondrial network architecture or intracellular compartmentalization, which may influence local redox microdomains and ROS signaling in vivo. Direct measurement of matrix versus intermembrane space ROS production, and quantification of antioxidant enzyme activity, would help clarify the underlying mechanisms. Furthermore, while our study controls for methodological variability by using identical protocols across tissues and species, differences in age, health status, and physical activity between human donors and animal subjects may influence mitochondrial function and should be acknowledged. Human myocardial samples were obtained from patients undergoing cardiac surgery and therefore may not fully represent non-diseased myocardium. However, the inclusion of skeletal muscle samples from healthy human volunteers, in which the same pattern of ROS production (predominance of RET CI over III_Qo_) was observed, supports the notion that the identified species-specific differences are not solely attributable to underlying disease or treatment effects. In addition, skeletal muscle samples were obtained exclusively from male subjects to reduce biological variability; therefore, potential sex-related differences in mitochondrial function and ROS production were not assessed and may limit the generalizability of our findings. It should also be noted that, under the substrate conditions used here (pyruvate, malate, and glutamate), rotenone can stimulate ROS production from several matrix dehydrogenases in addition to complex I [61]. In the present study, these signals were not separated, and for simplicity, we collectively refer to the rotenone-induced ROS as FET CI.

## 5. Conclusions

In summary, rat cardiac and skeletal muscle tissues produce more mitochondrial ROS than human tissues via the three mechanisms studied, consistent with higher respiratory capacity. However, when normalized to respiration, human mitochondria exhibit a greater relative capacity for ROS production at complex I, while maintaining lower output at complex III. These differences between human and rat tissues should be considered when extrapolating findings from rodent models, particularly in physiological and pathological processes where oxidative stress and redox signaling play key roles.

## Figures and Tables

**Table 1:** Cardiac patients’ characteristics.

	n = 12
Age (years)	64 ± 6
**Clinical characteristics**	
EuroScore II (%)	3.0 ± 2.0
LVEF (%)	54 ± 13
BMI (kg/m^2^)	26.5 ± 3.2
Hypertension, n (%)	9 (75)
Diabetes mellitus, n (%)	8 (67)
Cholesterol (mmol/L)	5.0 ± 0.8
HDL (mmol/L)	1.3 ± 0.1
LDL (mmol/L)	2.7 ± 1.0
TG (mmol/L)	2.3 ± 1.1
**Medications, n (%)**	
acetylsalicylic acid	9 (75)
antiplatelet therapy	2 (17)
anticoagulant therapy	6 (50)
beta blocker	9 (75)
ACE inhibitor/ARB	6 (50)
statin	10 (83)
diuretic	4 (33)
Ca-channel blocker	2 (17)
amiodarone	3 (25)
insulin	2 (17)
oral hypoglycemic agent	8 (67)

Data are presented as mean ± SD.

**Table 2:** Chemicals used for the assessment of respiration and ROS production.

Pyruvate	5 mM
Malate	0.5 mM
Glutamate	5 mM
Succinate	10 mM
ADP	2.5 mM
FCCP	titration in 0.1 μM steps
Rotenone	0.5 μM
Antimycin A	2.5 μM
Myxothiazol	2.5 μM
Malonate	1–14 mM
Oligomycin	100 nM
Amplex UltraRed	2.5 μM
HRP	1 U/mL
SOD	5 U/mL
H_2_O_2_	0.1 μM

ADP—adenosine diphosphate, FCCP—carbonylcyanide-p-trifluoromethoxyphenylhydrazone, HRP—horseradish peroxidase, SOD—superoxide dismutase, H_2_O_2_—hydrogen peroxide.

**Table 3:** Respiratory rates (pmol O_2_/(s∙mg)) measured under different substrate conditions in the four analyzed tissues.

Tissue	n	LEAK CI	LEAK CI + CII	OxPhos CI	OxPhos CI + CII
Human heart	12	3.3 ± 2.0 *	13.0 ± 3.0 *^#^	49.0 ± 9.5 *^#^	80.9 ± 17.7 *^#^
Rat heart	15	9.0 ± 2.6 ^#^	43.6 ± 5.8 ^#^	162.2 ± 34.7 ^#^	265.1 ± 45.4 ^#^
Human skeletal muscle	6	1.7 ± 0.5 *	5.1 ± 1.5 *	14.9 ± 4.9 *	28.1 ± 10.5 *
Rat skeletal muscle	9	4.8 ± 2.1	14.2 ± 3.0	34.4 ± 10.7	58.7 ± 15.1

Data are presented as mean ± SD. * *p* < 0.05 vs. the corresponding respiratory state in matched rat tissue; ^#^ *p* < 0.05 vs. the corresponding respiratory state in skeletal muscle within the same species.

## Data Availability

The data presented in this study are available on request from the corresponding author due to ethical restrictions.

## References

[1] Yang H.-M. (2025). Mitochondrial Dysfunction in Cardiovascular Diseases. Int. J. Mol. Sci..

[2] Liu M., Liu H., Dudley S.C. (2010). Reactive Oxygen Species Originating from Mitochondria Regulate the Cardiac Sodium Channel. Circ. Res..

[3] Münzel T., Camici G.G., Maack C., Bonetti N.R., Fuster V., Kovacic J.C. (2017). Impact of Oxidative Stress on the Heart and Vasculature. J. Am. Coll. Cardiol..

[4] Holmström K.M., Finkel T. (2014). Cellular mechanisms and physiological consequences of redox-dependent signalling. Nat. Rev. Mol. Cell Biol..

[5] Ristow M., Zarse K., Oberbach A., Klöting N., Birringer M., Kiehntopf M., Stumvoll M., Kahn C.R., Blüher M. (2009). Antioxidants prevent health-promoting effects of physical exercise in humans. Proc. Natl. Acad. Sci. USA.

[6] Hernansanz-Agustín P., Enríquez J.A. (2021). Generation of Reactive Oxygen Species by Mitochondria. Antioxidants.

[7] Santos A.L., Sinha S., Lindner A.B. (2018). The Good, the Bad, and the Ugly of ROS: New Insights on Aging and Aging-Related Diseases from Eukaryotic and Prokaryotic Model Organisms. Oxidative Med. Cell. Longev..

[8] Nakamura H., Takada K. (2021). Reactive oxygen species in cancer: Current findings and future directions. Cancer Sci..

[9] Goncalves R.L.S., Wang Z.B., Riveros J.K., Parlakgül G., Inouye K.E., Lee G.Y., Fu X., Saksi J., Rosique C., Hui S.T. (2025). CoQ imbalance drives reverse electron transport to disrupt liver metabolism. Nature.

[10] Sarnataro R., Velasco C.D., Monaco N., Kempf A., Miesenböck G. (2025). Mitochondrial origins of the pressure to sleep. Nature.

[11] Țocu G., Ștefănescu B.I., Stavăr Matei L., Țocu L. (2025). Phagocyte NADPH Oxidase NOX2-Derived Reactive Oxygen Species in Antimicrobial Defense: Mechanisms, Regulation, and Therapeutic Potential—A Narrative Review. Antioxidants.

[12] Bertero E., Maack C. (2018). Calcium Signaling and Reactive Oxygen Species in Mitochondria. Circ. Res..

[13] Dröse S., Brandt U. (2012). Molecular Mechanisms of Superoxide Production by the Mitochondrial Respiratory Chain. Mitochondrial Oxidative Phosphorylation.

[14] Brand M.D. (2016). Mitochondrial generation of superoxide and hydrogen peroxide as the source of mitochondrial redox signaling. Free. Radic. Biol. Med..

[15] Robb E.L., Hall A.R., Prime T.A., Eaton S., Szibor M., Viscomi C., James A.M., Murphy M.P. (2018). Control of mitochondrial superoxide production by reverse electron transport at complex I. J. Biol. Chem..

[16] Dubouchaud H., Walter L., Rigoulet M., Batandier C. (2018). Mitochondrial NADH redox potential impacts the reactive oxygen species production of reverse Electron transfer through complex I. J. Bioenerg. Biomembr..

[17] Chouchani E.T., Pell V.R., Gaude E., Aksentijević D., Sundier S.Y., Robb E.L., Logan A., Nadtochiy S.M., Ord E.N.J., Smith A.C. (2014). Ischaemic accumulation of succinate controls reperfusion injury through mitochondrial ROS. Nature.

[18] Lambert A.J., Brand M.D. (2004). Inhibitors of the quinone-binding site allow rapid superoxide production from mitochondrial NADH:ubiquinone oxidoreductase (complex I). J. Biol. Chem..

[19] Pryde K.R., Hirst J. (2011). Superoxide is produced by the reduced flavin in mitochondrial complex I: A single, unified mechanism that applies during both forward and reverse electron transfer. J. Biol. Chem..

[20] Dröse S., Brandt U. (2008). The mechanism of mitochondrial superoxide production by the cytochrome bc1 complex. J. Biol. Chem..

[21] Bleier L., Dröse S. (2013). Superoxide generation by complex III: From mechanistic rationales to functional consequences. Biochim. Biophys. Acta.

[22] Korge P., Calmettes G., John S.A., Weiss J.N. (2017). Reactive oxygen species production induced by pore opening in cardiac mitochondria: The role of complex III. J. Biol. Chem..

[23] Cavar M., Ljubkovic M., Bulat C., Bakovic D., Fabijanic D., Kraljevic J., Karanovic N., Dujic Z., Lavie C.J., Wisloff U. (2016). Trimetazidine does not alter metabolic substrate oxidation in cardiac mitochondria of target patient population. Br. J. Pharmacol..

[24] Lacomis D. (2000). The use of percutaneous needle muscle biopsy in the diagnosis of myopathy. Curr. Rheumatol. Rep..

[25] Kraljevic J., Marinovic J., Pravdic D., Zubin P., Dujic Z., Wisloff U., Ljubkovic M. (2013). Aerobic interval training attenuates remodelling and mitochondrial dysfunction in the post-infarction failing rat heart. Cardiovasc. Res..

[26] MacIntosh B.R., Esau S.P., Holash R.J., Fletcher J.R. (2011). Procedures for rat in situ skeletal muscle contractile properties. J. Vis. Exp..

[27] Larsen S., Kraunsøe R., Gram M., Gnaiger E., Helge J.W., Dela F. (2014). The best approach: Homogenization or manual permeabilization of human skeletal muscle fibers for respirometry?. Anal. Biochem..

[28] Makrecka-Kuka M., Krumschnabel G., Gnaiger E. (2015). High-Resolution Respirometry for Simultaneous Measurement of Oxygen and Hydrogen Peroxide Fluxes in Permeabilized Cells, Tissue Homogenate and Isolated Mitochondria. Biomolecules.

[29] Grivennikova V.G., Vinogradov A.D. (2013). Partitioning of superoxide and hydrogen peroxide production by mitochondrial respiratory complex I. Biochim. Biophys. Acta.

[30] Li Puma L.C., Hedges M., Heckman J.M., Mathias A.B., Engstrom M.R., Brown A.B., Chicco A.J. (2020). Experimental oxygen concentration influences rates of mitochondrial hydrogen peroxide release from cardiac and skeletal muscle preparations. Am. J. Physiol.-Regul. Integr. Comp. Physiol..

[31] Komlódi T., Sobotka O., Gnaiger E. (2021). Facts and artefacts on the oxygen dependence of hydrogen peroxide production using Amplex UltraRed. Bioenerg. Commun..

[32] Goncalves R.L.S., Quinlan C.L., Perevoshchikova I.V., Hey-Mogensen M., Brand M.D. (2015). Sites of superoxide and hydrogen peroxide production by muscle mitochondria assessed ex vivo under conditions mimicking rest and exercise. J. Biol. Chem..

[33] Barth E., Stämmler G., Speiser B., Schaper J. (1992). Ultrastructural quantitation of mitochondria and myofilaments in cardiac muscle from 10 different animal species including man. J. Mol. Cell. Cardiol..

[34] Park S.-Y., Gifford J.R., Andtbacka R.H.I., Trinity J.D., Hyngstrom J.R., Garten R.S., Diakos N.A., Ives S.J., Dela F., Larsen S. (2014). Cardiac, skeletal, and smooth muscle mitochondrial respiration: Are all mitochondria created equal?. Am. J. Physiol.-Heart Circ. Physiol..

[35] Hou C., Metcalfe N.B., Salin K. (2021). Is mitochondrial reactive oxygen species production proportional to oxygen consumption? A theoretical consideration. BioEssays.

[36] Fang T., Chan A., Chew-Harris J., Pham T. (2026). Distinct profiles of mitochondrial bioenergetics and redox balance in left atrial and ventricular myocardium in the healthy rat heart. Exp. Physiol..

[37] Escala A. (2022). Universal relation for life-span energy consumption in living organisms: Insights for the origin of aging. Sci. Rep..

[38] Sahlin K., Shabalina I.G., Mattsson C.M., Bakkman L., Fernström M., Rozhdestvenskaya Z., Enqvist J.K., Nedergaard J., Ekblom B., Tonkonogi M. (2010). Ultraendurance exercise increases the production of reactive oxygen species in isolated mitochondria from human skeletal muscle. J. Appl. Physiol..

[39] Galganski L., Wojcicki K., Jarmuszkiewicz W., Zoladz J.A. (2025). Impact of endurance training on mitochondrial H_2_O_2_ production and NRF2 levels in different rat organs. Front. Mol. Biosci..

[40] Gusdon A.M., Fernandez-Bueno G.A., Wohlgemuth S., Fernandez J., Chen J., Mathews C.E. (2015). Respiration and substrate transport rates as well as reactive oxygen species production distinguish mitochondria from brain and liver. BMC Biochem..

[41] Jedlička J., Tůma Z., Razak K., Kunc R., Kala A., Proskauer Pena S., Lerchner T., Ježek K., Kuncová J. (2022). Impact of aging on mitochondrial respiration in various organs. Physiol. Res..

[42] Chen Q., Vazquez E.J., Moghaddas S., Hoppel C.L., Lesnefsky E.J. (2003). Production of reactive oxygen species by mitochondria: Central role of complex III. J. Biol. Chem..

[43] Muller F.L., Liu Y., Van Remmen H. (2004). Complex III releases superoxide to both sides of the inner mitochondrial membrane. J. Biol. Chem..

[44] Treberg J.R., Quinlan C.L., Brand M.D. (2010). Hydrogen peroxide efflux from muscle mitochondria underestimates matrix superoxide production—A correction using glutathione depletion. FEBS J..

[45] Munro D., Pamenter M.E. (2019). Comparative studies of mitochondrial reactive oxygen species in animal longevity: Technical pitfalls and possibilities. Aging Cell.

[46] Yin Z., Burger N., Kula-Alwar D., Aksentijević D., Bridges H.R., Prag H.A., Grba D.N., Viscomi C., James A.M., Mottahedin A. (2021). Structural basis for a complex I mutation that blocks pathological ROS production. Nat. Commun..

[47] Lopez-Fabuel I., Le Douce J., Logan A., James A.M., Bonvento G., Murphy M.P., Almeida A., Bolaños J.P. (2016). Complex I assembly into supercomplexes determines differential mitochondrial ROS production in neurons and astrocytes. Proc. Natl. Acad. Sci. USA.

[48] Lu J., Holmgren A. (2014). The thioredoxin antioxidant system. Free Radic. Biol. Med..

[49] Ströher E., Millar A.H. (2012). The biological roles of glutaredoxins. Biochem. J..

[50] Wang Y., Branicky R., Noë A., Hekimi S. (2018). Superoxide dismutases: Dual roles in controlling ROS damage and regulating ROS signaling. J. Cell Biol..

[51] Munro D., Baldy C., Pamenter M.E., Treberg J.R. (2019). The exceptional longevity of the naked mole-rat may be explained by mitochondrial antioxidant defenses. Aging Cell.

[52] Chouchani E.T., Pell V.R., James A.M., Work L.M., Saeb-Parsy K., Frezza C., Krieg T., Murphy M.P. (2016). A Unifying Mechanism for Mitochondrial Superoxide Production during Ischemia-Reperfusion Injury. Cell Metab..

[53] Mottahedin A., Prag H.A., Dannhorn A., Mair R., Schmidt C., Yang M., Sorby-Adams A., Lee J.J., Burger N., Kulaveerasingam D. (2023). Targeting succinate metabolism to decrease brain injury upon mechanical thrombectomy treatment of ischemic stroke. Redox Biol..

[54] Marinovic J., Mihanovic I., Maltar-Strmecki N., Bulat C., Zanchi J., Ljubkovic M. (2021). Coronary collateralization prevents myocardial ROS surge induced by revascularization after non-ST-elevation acute coronary syndrome: A pilot study. Prog. Cardiovasc. Dis..

[55] Kohlhauer M., Dawkins S., Costa A.S.H., Lee R., Young T., Pell V.R., Choudhury R.P., Banning A.P., Kharbanda R.K., Saeb-Parsy K. (2018). Metabolomic Profiling in Acute ST-Segment–Elevation Myocardial Infarction Identifies Succinate as an Early Marker of Human Ischemia–Reperfusion Injury. J. Am. Heart Assoc..

[56] Penna C., Comità S., Tullio F., Alloatti G., Pagliaro P. (2022). Challenges facing the clinical translation of cardioprotection: 35 years after the discovery of ischemic preconditioning. Vasc. Pharmacol..

[57] Pagliaro P., Penna C., Femminò S., Welt F.G.P. (2026). Insights in ischemia/reperfusion injury and cardioprotection: Neglected and emerging pathways and therapeutic targets for a personalized therapy. Basic Res. Cardiol..

[58] AlAhmad M., Isbea H., Shitaw E., Li F., Sivaprasadarao A. (2024). NOX2-TRPM2 coupling promotes Zn^2+^ inhibition of complex III to exacerbate ROS production in a cellular model of Parkinson’s disease. Sci. Rep..

[59] Zotta A., Toller-Kawahisa J., Palsson-McDermott E.M., O’Carroll S.M., Henry Ó.C., Day E.A., McGettrick A.F., Ward R.W., Ryan D.G., Watson M.A. (2025). Mitochondrial respiratory complex III sustains IL-10 production in activated macrophages and promotes tumor-mediated immune evasion. Sci. Adv..

[60] Rottenberg H., Covian R., Trumpower B.L. (2009). Membrane Potential Greatly Enhances Superoxide Generation by the Cytochrome bc1 Complex Reconstituted into Phospholipid Vesicles. J. Biol. Chem..

[61] Quinlan C.L., Goncalves R.L.S., Hey-Mogensen M., Yadava N., Bunik V.I., Brand M.D. (2014). The 2-Oxoacid Dehydrogenase Complexes in Mitochondria Can Produce Superoxide/Hydrogen Peroxide at Much Higher Rates Than Complex I. J. Biol. Chem..

